# Design and Fabrication of Fiber-Optic Nanoprobes for Optical Sensing

**DOI:** 10.1007/s11671-010-9744-5

**Published:** 2010-08-31

**Authors:** Yan Zhang, Anuj Dhawan, Tuan Vo-Dinh

**Affiliations:** 1 Fitzpatrick Institute for Photonics, Duke University, Durham, NC 27708, USA; 2 Department of Biomedical Engineering, Duke University, Durham, NC 27708, USA; 3 Department of Chemistry, Duke University, Durham, NC 27708, USA

**Keywords:** Fiber-optic nanoprobes, Focused ion beam, Shadow evaporation, Optical sensing

## Abstract

This paper describes the design and fabrication of fiber-optic nanoprobes developed for optical detection in single living cells. It is critical to fabricate probes with well-controlled nanoapertures for optimized spatial resolution and optical transmission. The detection sensitivity of fiber-optic nanoprobe depends mainly on the extremely small excitation volume that is determined by the aperture sizes and penetration depths. We investigate the angle dependence of the aperture in shadow evaporation of the metal coating onto the tip wall. It was found that nanoaperture diameters of approximately 50 nm can be achieved using a 25° tilt angle. On the other hand, the aperture size is sensitive to the subtle change of the metal evaporation angle and could be blocked by irregular metal grains. Through focused ion beam (FIB) milling, optical nanoprobes with well-defined aperture size as small as 200 nm can be obtained. Finally, we illustrate the use of the nanoprobes by detecting a fluorescent species, benzo[a]pyrene tetrol (BPT), in single living cells. A quantitative estimation of the numbers of BPT molecules detected using fiber-optic nanoprobes for BPT solutions shows that the limit of detection was approximately 100 molecules.

## Introduction

The emergence of nanotechnology opens new horizons for nanosensors and nanoprobes that are suitable for intracellular measurements. Nanosensors provide critical information for monitoring biomolecular processes within a single living cell, thus could provide great advances in biomedical research and clinical applications. Fiber-optic nanosensors with nanoscale dimensions are capable of sensing intracellular/intercellular physiological and biological parameters in submicron environments. Tapered fibers with distal diameters between 20 and 500 nm have been demonstrated for near-field scanning optical microscopy (NSOM) [1,2]. Chemical nanosensors were developed for monitoring calcium and nitric oxide, among other physico-chemicals in single cells [3,4]. Vo-Dinh and coworkers have developed nanobiosensors to detect biochemical targets inside living single cells [5-12]. Fiber-optic nanoprobe promises to be an area of growing research that could potentially provide an imaging tool for monitoring individual cells and even biological molecules. Single-molecule detection and imaging schemes using nanofibers could open new possibilities in the investigation of the complex biochemical reactions and pathways in biological and cellular systems leading to important applications in medicine and health effect studies.

Optical nanotips were first developed as scanning probes in near-field optical microscopes [2]. Such nanoprobes can achieve resolution as high as λ/50, where λ is the wavelength of light [1]. It is important to control aperture size, taper shape, and metal coating to achieve a better performance [13]. The fiber-optic probes were fabricated by laser-heated pulling or chemical etching [14-16]. Laser-pulled fiber tips can achieve diameters smaller than 50 nm with small cone angles [14]. Chemical etching tips have larger cone angles and similar apex sizes [15]. However, it is often difficult to control the etching process. The side of the fiber was further coated with silver, aluminum, or gold films to confine the light [9,14,17]. Traditional manufacturing processes still limit the quality of metal-coated fiber probes. Optical throughput of pulled nanoprobes is limited by the sharp taper angle. Chemical-etched tips have higher throughput; however, they do not have a flat distal end as laser-pulled ones which are difficult to form well-defined nanoapertures in shadow evaporation. Moreover, shadow evaporation often leads to either complete or irregular coated tip. Grainy structures of metal thin film increase the distance between the aperture and the sample, which reduce the resolution and intensity. It is also easy to form pin holes at the tapered region that could cause light-leaking. The aperture deviates from ideal circular shape because of grains. A quantitative analysis of probe transmission efficiency becomes difficult. Focused ion beam (FIB) fabrication of nanostructures has been applied on optical fibers for chemical sensing [18]. FIB milling for nanostructure formation allows precise control of size and shape in nanometer accuracy. This paper deals specifically with the metal coating on the formation of nanoaperture at the tip end. Coating materials and angles greatly affect the quality of the nanoprobe. By combining with focused ion beam milling, nanoprobes with well-defined aperture as small as 200 nm have been obtained. We investigate the capacity of the nanoprobes by detect benzo[a]pyrene tetrol in living cells.

## Experimental Procedures

### Nanoprobe Fabrication

**Figure 1:**
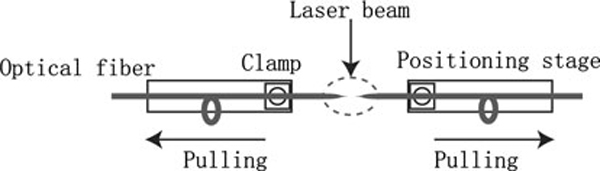
**Fabrication of nanofibers by laser pulling**.

Optical nanoprobes were fabricated through laser pulling method, which consists of local heating of an optical fiber (Polymicro Technologies FVP400440480) using a laser and subsequently pulling the fiber apart. Fabrication of nanosensors requires techniques capable of making reproducible optical fibers with submicron-size-diameter core. Figure 1 illustrates the experimental setup for the fabrication of nanofibers using the micropipette puller (Sutter Instruments P-2000) [9]. As the laser pulling process is a time-dependent heating effect, laser power, timing of pulling, velocity setting, and pulling force all contribute to the taper shape and tip size. Since transmission efficiency is highly dependent on the taper shape, it is crucial to control the tip shape in the fabrication of high-quality nanoprobes.

The sidewall of the tapered end was then coated with a thin layer of metal, such as silver, aluminum, or gold to prevent light leakage of the excitation light on the tapered side of the fiber. An array of fiber probes was attached on a rotating motor inside a thermal evaporation chamber (Quorum Technologies E6700). The rotation rate was controlled by a microcontroller board (Parallax). While the probes were rotating, the metal was allowed to evaporate onto the tapered side of the fiber tip to form a thin coating. The nanoaperture was formed through shadowed evaporation as the fibers were tilting away from the source. The nanoprobes were characterized with scanning electron microscopy (FEI XL30).

In order to fabricate well-defined fiber-optic nanoprobe tips, we employed focused ion beam (FIB) milling of nanoapertures in the metallic films deposited on tapered tips of optical fibers. Before carrying out FIB milling, the optical fibers were coated with metallic films (aluminum, silver or gold) using electron beam evaporation (CHA Industries Solution E-Beam). During the evaporation process, the fiber-optic tips faced the metal source to ensure that the fiber side walls and the tips were completely covered with a thin metallic layer (100–150 nm). The sample mount was rotated to improve uniformity and the thickness of the metallic film was monitored by a quartz crystal monitor. The deposition rate was varied between 0.05 and 0.2 nm s^-1^ at a chamber pressure of ~3 × 10^-6^ Torr for the electron beam evaporated films.

A Hitachi FB2100 focused ion beam milling machine with a gallium ion source was used to fabricate the nanoapertures on the fiber tips. Beam currents and accelerating voltages of 0.01 nA and 40 keV energy were typically used. The desired nanostructures were milled by rastering the ion beam and employing a beam blanker. The beam blanker shuts on and off according to a 8-bit grayscale, 512 by 512 pixel image file. Tapered optical fiber tips with nanoapertures were fabricated by employing FIB milling at magnifications varying between 3000× and 18000× depending on the desired minimum aperture size. To form metallic nanostructures on the tips of optical fibers, a special fiber holder that could fit in the FIB stage was fabricated.

### Optical Measurement

**Figure 2:**
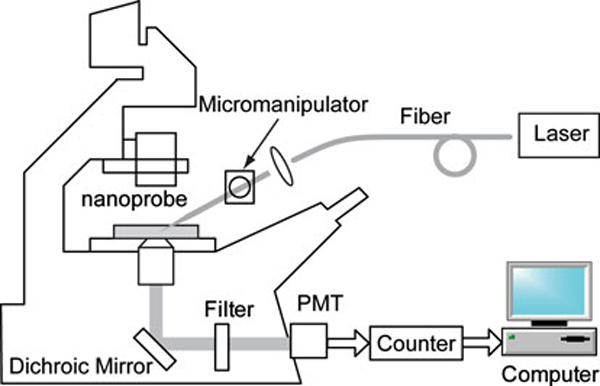
**Instrumental system for fluorescence measurements using nanoprobes**.

The optical measurement system used for nanoprobe is schematically illustrated in Figure 2. For nanoprobe measurements, the 325 nm line of a HeCd laser (CVI Melles Griot, 15 mW laser power) was focused onto a 400 μm delivery fiber. A tapered fiber was coupled to the delivery fiber through a capillary tubing and was secured to the micromanipulators (Narishige MLW-3) on a Zeiss Axiovert 200M microscope (Zeiss). The fluorescence emitted from the region beyond the aperture was collected by the microscope objective and passed through a bandpass filter (386 nm) and then focused onto a photomultiplier tube (PMT, Hamamatsu, HC125-2) for detection. The output from the PMT was recorded on a universal counter (Agilent 53131A), and a personal computer (PC) was used for further data treatment.

Nanoprobes were also used to investigate BPT in single cells. PC3 human prostate cancer cells were incubated with 1 μM BPT in PBS for 2 h. Control cells are treated with PBS only. All dishes were rinsed with PBS prior to measurement. Nanoprobes were controlled by the micromanipulator to puncture the cell and keep inside while taking the measurement.

## Results and Discussion

### Effect of the Metal Evaporation Angle

**Figure 3:**
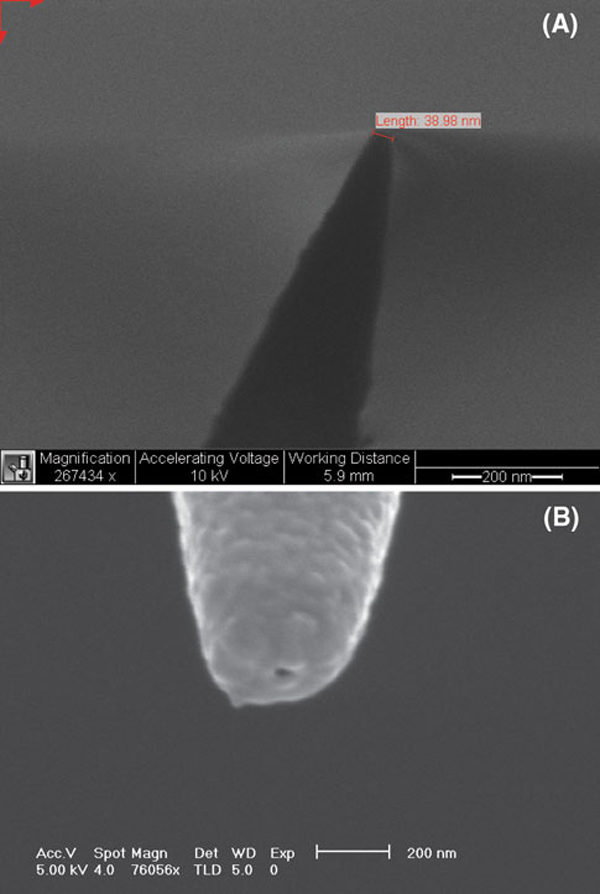
**SEM images of a an uncoated nanofiber and b a gold-coated nanofiber**.

A scanning election microscopy (SEM) photograph of one of the nanofibers fabricated by the laser pulling method is shown in Figure 3a. The distal end of the nanofiber is approximately 40 nm. The fiber was pointing away from the evaporation source with an angle of approximately 25°. The tapered end was coated with ~75–100 nm of metal in the thermal evaporator. With the metal coating, the size of the probe tip is approximately 200–250 nm (Figure 3b).

**Figure 4:**
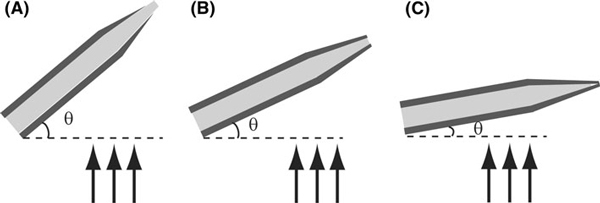
**Nanoaperture formation by shadow evaporation with a high angle (>25°), b medium angle (~25°), and c low angle (<25°)**.

**Figure 5:**
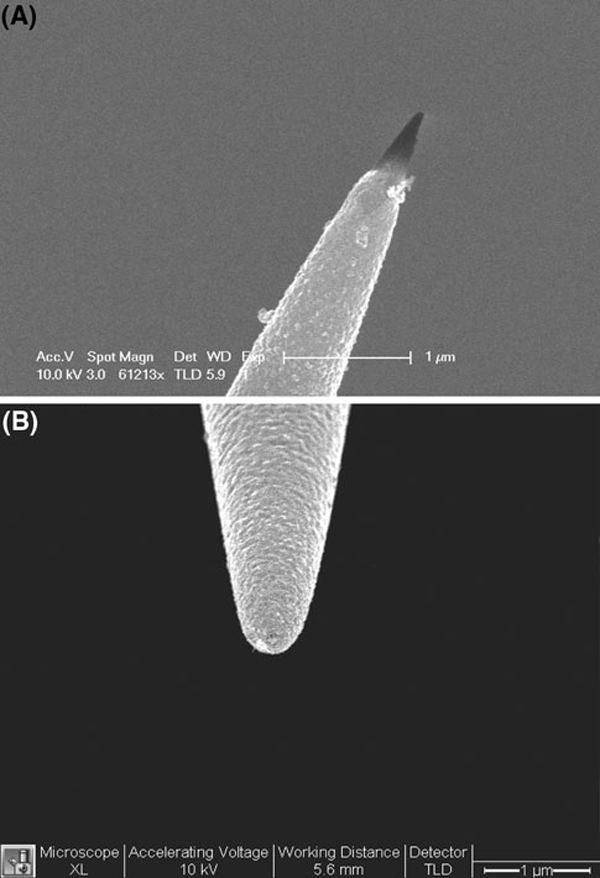
**SEM images of silver-coated nanofibers under different angles: a 40° and b 20°**.

Due to the inclination angle, the tip ends are shadowed from evaporation when the fiber tips are tilted away from the source. The effect of shadow evaporation angle is illustrated in Figure 4. A nanoaperture was formed on the tip end for optical excitation. The size of the nanoaperture is related to the angle between fiber axis and evaporation direction. For example, if the angle is less than 20°, most of the fibers are fully covered with metal and no aperture is visible using SEM. On the contrary, if the angle is higher than 30°, a larger area of the distal end of the fiber tip will be exposed (Figure 5). The optimal angle of inclination can be determined through characterization of nanoapertures under SEM. The SEM can determine the actual size of the tip aperture. However, having a nanometer-sized aperture does not guarantee a good near-field probe. The tip aperture, even though it may appear small on the SEM, could be a result of aluminum over-coating, and hence not be a functional light aperture for actual measurements. Therefore, a functional scan is necessary to reveal the near-field effect from the probe. Near-field scanning optical microscope (NSOM) enables functional analysis of the nanosensor probe by performing a scan on a standard sample, e.g., a Fischer pattern. The standard sample usually consists of patterns with size less than the diffraction limits (0.5 λ) that can be determined by an atomic force microscope. The nanosensor probe was attached to an NSOM system working as an NSOM probe. Typically, the aperture of the probe roughly determines the resolution of the image. In other word, the image quality thus represents the quality of the probe.

### Effect of the Different Metal Coatings on the Nanoprobes

The metallic coating process is a critical step in nanoprobe fabrication. A thin film of an optically opaque metal such as aluminum, silver, or gold is coated along the outside walls of the tapered optical fiber tip to form an optical light pipe free of defects, which would permit photons to escape from the tapered sides of the optical fiber. An optical aperture to allow evanescent wave excitation is formed at the tip's apex by angled evaporation. Silver has been used in nanoprobe fabrication [9]. It has high reflectivity in the visible and IR range and very stable in aqueous solutions as long as oxidizing agents or complexing agents are not present. But a silver layer will oxidize rapidly under ordinary atmospheric conditions and will not exhibit a high reflectance below 400 nm. Therefore, it is desirable to use the nanoprobe right after metal evaporation. Otherwise, the light shielding will deteriorate or even the coating will peel off after a few days in air.

Gold thin film was demonstrated to be a very stable coating under environmental conditions although it does not have high reflectance in visible range. An interface layer such as Cr is required to increase adhesion between gold and the fiber silica surface. Gold has a high melting temperature (660°C for Al, 960°C for Ag, and 1,060°C for Au) and a good thermal resistance. The thermal stress generated during metallic film deposition damages the aperture due to very different thermal expansion coefficients of metal and quartz. Gold coating has the lowest thermal expansion coefficient (23.1 × 10^-6^/°C for Al, 18.9 × 10^-6^/°C for Ag, 14.2 × 10^-6^/°C for Au, 0.55 × 10^-6^/°C for SiO_2_), which will reduce the thermal destruction of the fiber tip.

**Figure 6:**
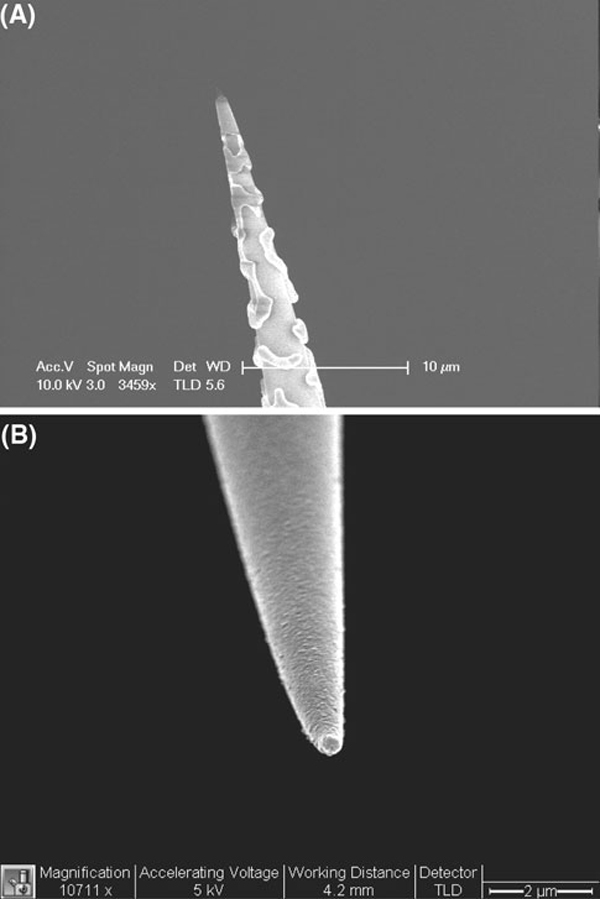
**SEM images of a silver- and b aluminum-coated nanofibers after plasma cleaning**.

Aluminum is a desirable material to use because it has the highest extinction coefficient of all metals. Aluminum adheres to fibers more firmly than silver or gold so that no interface layer is required and general cleaning does not affect the coating. Figure 6 compares the nanofibers after argon plasma cleaning (Emitech K-1050X, 100 W, 5 min). Silver coatings are easily peeled off while aluminum coatings exhibit no changes under the same condition. Aluminum is inert toward corrosive agents since a protective oxide layer is formed readily upon contact to the air. However, it is difficult to evaporate aluminum as a thin film while maintaining smooth films with small grain sizes [19]. Grainy films contribute to the high background in near-field sensing. The grain diameter is highly and sensitively dependent on the deposition pressure. Below 5 × 10^-6^ torr, the size of the individual grains is smaller than 100 nm. There was a relationship between rate of metallic deposition and subsequent surface roughness, and studies revealed that higher coating rate (>10 nm/s) resulted in better smoothness and the film opacity required for our intended sensor applications.

### Nanoprobe Fabrication using Focus Ion Beam (FIB) Milling

**Figure 7:**
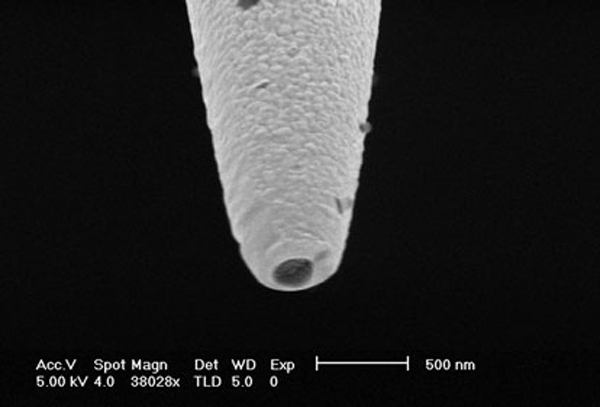
**FIB-etched nanoprobe with aperture diameter of 200 nm**.

The first FIB milling process involved placing the metal-coated optical fiber tips horizontally, i.e., orthogonal to the focused ion beam and then cutting the tips (both the tapered silica fiber and the metal over-coating) such that an aperture could be developed at the tip. Milling of the nanoapertures using this process has an advantage that it is not time-consuming as several tips placed adjacent to each other can be cut with the same beam raster, it gives reliable nanoprobes with well-defined nanoapertures of circular geometry, and the length of the optical fiber nanoprobes can be longer, which can make coupling of light into the optical fibers easier. The second process involved positioning the fiber tips such that they faced the focused ion beam and then carrying out the milling of the nanoapertures at the tip. Although this process enables fabrication of nanoapertures of different geometries and sizes in a very controllable manner, it limits the length of the fiber-optic probe as only a certain length of the optical fiber probe can be placed vertically in the Hitachi FB2100 focused ion beam milling machine. By milling with a focused ion beam, an aperture with controllable shape and diameter as small as 200 nm was achieved (Figure 7). The angle of evaporation is not necessary in FIB, therefore reducing the chance of pin-hole formation. A clean aperture free from grains also facilitates the subsequent functionalization of bioreceptor molecules on the fiber distal end for biosensing applications. FIB processing is a promising technique in nanoprobe fabrication in addition to laser pulling and chemical etching.

### Fluorescence Measurement of Benzo[a]Pyrene Tetrol (BPT) Using Nanoprobes

**Figure 8:**
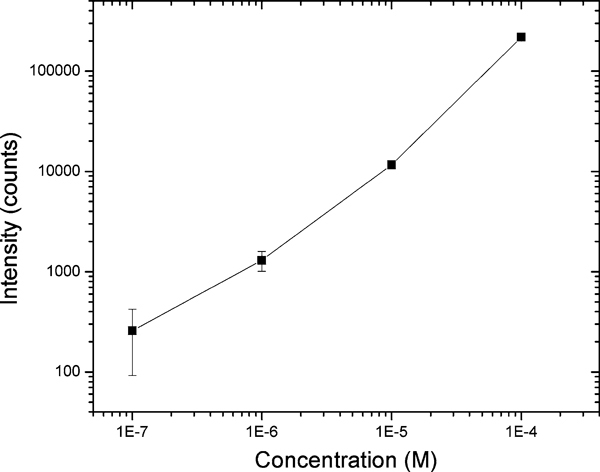
**Fluorescence intensities of benzo[a]pyrene tetrol (BPT) measured with a fiber-optic nanoprobe**.

**Figure 9:**
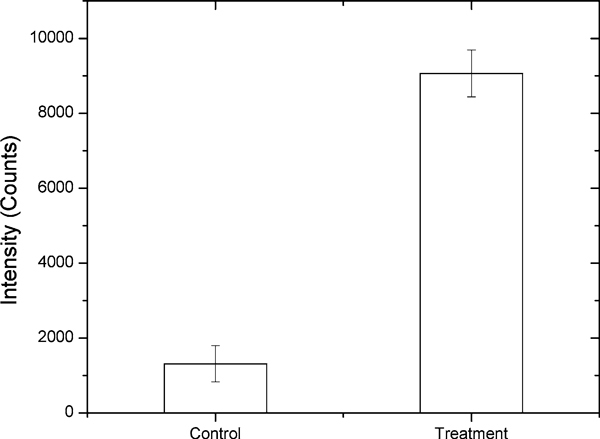
**Intracellular measurement of benzo[a]pyrene tetrol (BPT) with nanoprobes**. PC3 human prostate cancer cells were incubated with 1 μM BPT in PBS for 2 h. Control cells are treated with PBS only. All *dishes* were rinsed with PBS prior to measurement.

Chemical analysis of polynuclear aromatic hydrocarbons (PAHs) is of great environmental and toxicological interest because many of them have been shown to be mutagens and/or potent carcinogens in laboratory animal assays. Benzo[a]pyrene (BaP), which has been extensively investigated, is one of the more potent carcinogens among PAHs and is a fundamental indicator of exposure and carcinogenic activity of all PAHs. In order to facilitate the study of intracellular dynamics of benzo[a]pyrene tetrol (BPT), the related biomarker under BaP exposure, a quantitative estimation of the numbers BPT molecules detected using fiber-optic nanoprobes for solutions containing different BPT concentrations was performed and shown in Figure 8. The limit of detection that corresponds to the amount of analyte emitting a signal 3 times the standard deviation of the noise was determined to be 1 μM for BPT. Under 1 μM, the dark count noise from PMT was stronger than the signal. The detection volume can be estimated as 17 aL for 200 nm aperture probe based on Bethe–Bouwkamp theory. Therefore, the limit of detection was approximately 100 BPT molecules. Figure 9 shows the intracellular measurement of BPT in PC3 human prostate cancer cells. The cells were incubated with 1 μM BPT in PBS for 2 h at 37°C. Control cells are PC3 cells treated with PBS only. All dishes were rinsed with PBS prior to measurement. It is apparent that the treated cells exhibited higher fluorescence reading than the control group. Although in our preliminary experiments the living cells were directly incubated with BPT, the results illustrate that the nanoprobes can be employed to detect very low concentrations of fluorescent species such as BPT molecules that are important biomarkers of exposure and carcinogenic activity of related PAHs, inside living cells.

## Conclusion

Fiber-optic nanoprobes have opened up new applications in molecular biology and medical diagnostics. Due to their small sizes, nanosensor provides important tools for minimal invasive analysis at single cellular or sub-cellular level. Because transmission efficiency is highly related to the aperture size, control the nanoaperture size is essential in the fabrication of high-quality nanoprobes. Subtle changes in the tilt angle during metal evaporation can greatly affect the size or even the existence of the aperture. A much more rational fabrication process would involve a nanofabrication technique such as FIB, in which aperture size could be independently controlled from evaporation. The detection sensitivity of fiber-optic nanoprobes depends mainly on the extremely small excitation or detection volume set by the aperture sizes and penetration depths. This effectively reduces background fluorescence, thereby enhance detection sensitivity. Nanofabrication would also greatly improve the reproducibility of aperture shapes and hence the optical performance of near-field probes.
